# Colliers and Cardiff Hospitals

**Published:** 1912-12-28

**Authors:** 


### Colliers and Cardiff Hospitals.

### A CITY AND DISTRICT HOSPITAL SOCIETY FOUNDED.

The Cardiff and District Hospital Society has just been instituted, and its first Secretary appointed. The Society has been established and is being organised by a committee of working men, loyal and consistent friends of the King Edward VII.'s Hospital and representative of its working-men contributors, both in the city of Cardiff and the wide area served by the hospital, which includes the populous colliery district. The main principle of the scheme is the systematic contribution of a penny per week per member towards the funds of the Society. A fixed proportion of the moneys so contributed will be paid for the upkeep of the King Edward VII.'s Hospital, and out of the balance convalescent homes and kindred institutions will be supported for the benefit of the members.

The first Secretary of the Society is Mr. Rowland Evans, J.P., of Merthyr Vale. Mr. Evans has personal knowledge and experience of the industrial conditions of the district, as he has been a working collier for eighteen years, and for the past eight years has held the responsible position of check-weigher to the Merthyr Vale Colliery workmen, who number over 3,500. He is an Alderman of the Merthyr Borough Council and a Justice of the Peace, and was Mayor of Merthyr during the Coronation year. "No one is better qualified," writes a well-informed correspondent, "to explain, with eloquence and feeling, the humane objects of the Society to large bodies of men, and this Mr. Evans can do with equal facility in Welsh and English." The Society will keep in close touch with the hospital, and, by the unanimous choice of the working people themselves, Colonel Bruce Vaughan has been appointed Chairman, and Major-General Lee Treasurer. It has long been Colonel Vaughan's idea, we understand, to inaugurate a movement of this character to cover the whole area served by the hospital, but it has not been found possible to do so until his schemes for the equipment and extension of the hospital were completed.

